# Increased Susceptibility of *Gracilinanus microtarsus* Liver Mitochondria to Ca^2+^-Induced Permeability Transition Is Associated with a More Oxidized State of NAD(P)

**DOI:** 10.1155/2015/940627

**Published:** 2015-10-25

**Authors:** Juliana A. Ronchi, Barbara Henning, Felipe G. Ravagnani, Tiago R. Figueira, Roger F. Castilho, Sergio F. dos Reis, Anibal E. Vercesi

**Affiliations:** ^1^Department of Clinical Pathology, Faculty of Medical Sciences, State University of Campinas (UNICAMP), 13087-877 Campinas, SP, Brazil; ^2^Graduate Program in Ecology, Biology Institute, State University of Campinas, 13087-877 Campinas, SP, Brazil; ^3^Department of Animal Biology, Biology Institute, State University of Campinas, 13087-877 Campinas, SP, Brazil

## Abstract

In addition to be the cell's powerhouse, mitochondria also contain a cell death machinery that includes highly regulated processes such as the membrane permeability transition pore (PTP) and reactive oxygen species (ROS) production. In this context, the results presented here provide evidence that liver mitochondria isolated from *Gracilinanus microtarsus*, a small and short life span (one year) marsupial, when compared to mice, are much more susceptible to PTP opening in association with a poor NADPH dependent antioxidant capacity. Liver mitochondria isolated from the marsupial are well coupled and take up Ca^2+^ but exhibited a much lower Ca^2+^ retention capacity than mouse mitochondria. Although the known PTP inhibitors cyclosporin A, ADP, and ATP significantly increased the marsupial mitochondria capacity to retain Ca^2+^, their effects were much larger in mice than in marsupial mitochondria. Both fluorescence and HPLC analysis of mitochondrial nicotinamide nucleotides showed that both content and state of reduction (mainly of NADPH) were lower in the marsupial mitochondria than in mice mitochondria despite the similarity in the activity of the glutathione peroxidase/reductase system. Overall, these data suggest that PTP opening is an important event in processes of Ca^2+^ signalling to cell death mediated by mitochondrial redox imbalance in *G. microtarsus*.

## 1. Introduction

It is well established that Ca^2+^ modulates many vital processes through transient increases in its free concentrations in different cell compartments [1]. This includes several pathways of energy metabolism, synaptic transmission, gene expression, and cell survival or death [2–5].

In order to fulfill these physiological roles Ca^2+^ movements across plasma cell membrane are driven directly or indirectly by ATP hydrolysis; therefore, defects in processes of cellular ATP supply may lead to dysregulation in Ca^2+^ signaling that may compromise cell functioning [1, 4]. In regard to mechanisms of survival or death, evidence has been provided that intramitochondrial Ca^2+^ signals for both (i) the control of oxidative phosphorylation, required for cell function [3, 5, 6], and (ii) reactive oxygen generation, required for both survival and death [4, 7]. Indeed, it is now generally accepted that superoxide as well as other reactive oxygen species (ROS) can function both beneficially or adversely [4, 8]. At progressively increasing physiological levels they may successively regulate cellular processes such as proliferation and differentiation, activate adaptive programs such as transcriptional upregulation of antioxidant genes and, at higher levels, they may signal to senescence and regulated cell death [8]. Direct damaging effects of free radicals may only occur under extreme conditions [9, 10]. In addition to the physiological processes, it seems that mitochondrial oxidative stress is responsible for the development and progression of a series of pathologies such as cancer, diabetes, inflammatory diseases, hypertension, neurodegenerative, and ischemia-related diseases and aging [4, 11]. In this context, one event that may participate in all of these processes via the mitochondrial pathway of cell death, either by apoptosis or necrosis, is the so-called mitochondrial membrane permeability transition (MPT) [12, 13].

The state of MPT is characterized by the opening of a nonspecific inner membrane pore induced by the combination of high matrix [Ca^2+^] and oxidative stress [9, 13]. Considering the understanding of how Ca^2+^ and reactive oxygen act synergistically in the process of permeability transition pore (PTP) opening, evidence has been provided that mitochondria are more susceptible to MPT when their antioxidant systems, represented mainly by NADPH, are exhausted [9, 14, 15]. In fact, MPT can be induced by prooxidants and prevented or even reversed by antioxidants [13].

Altogether the above considerations indicate that mitochondria are multifunctional organelles that control the production of ATP, participate in intracellular Ca^2+^ homeostasis, and function as a main source of reactive oxygen. Therefore, it might be reasonable to consider that genetic variations or dysfunctions in any of these vital mitochondrial properties may intrinsically modify the susceptibility to many diseases and aging [16]. In this regard, the Brazilian gracile opossum (*Gracilinanus microtarsus*) is a short lifespan marsupial with high mortality associated with heightened levels of stress due to aggressive behavior during the mating period [17–22].* G. microtarsus* has a most unusual and remarkable reproductive pattern in that the majority of males die after the first mating period, a condition known as partial semelparity in evolutionary ecology [18, 23]. Available evidence suggests that the cost of reproduction is detrimental to the survival of* G. microtarsus* and is conceivably related to the short lifespan of this species in nature.

The aims of the present work were, firstly, the analysis of mitochondrial bioenergetics in isolated liver mitochondria from* G. microtarsus*, taking into consideration the possible role of these organelles as key players in lifespan regulation of this marsupial and, secondly, to verify whether MPT plays any role in the process.

## 2. Material and Methods

### 2.1. Chemicals

Most of the reagents used were obtained from Sigma-Aldrich. Calcium Green-5N hexapotassium salt was purchased from Invitrogen (Invitrogen, Carlsbad, CA, USA).

### 2.2. Animals

C57BL/6/JUnib mice were provided by the Campinas University Multidisciplinary Center for Biological Research in Laboratory Animals (CEMIB/UNICAMP, Campinas, Brazil). The C57BL/6/JUnib mice substrain does not carry the mutation in the nicotinamide nucleotide transhydrogenase (*Nnt*) gene [15] that affects the mitochondrial function of some other C57BL/6 mice substrains [24].* Nnt* is a well conserved gene and is present in marsupials (*Monodelphis domestica*, gene ID: 100012732). Mice were kept under standard laboratory conditions (20–22°C and 12 h/12 h light/dark cycle) with free access to a standard diet (Labina/Purina, Campinas, SP, Brazil) and tap water. Although mice are phylogenetically distant from marsupials, mouse features a similar body size to* G. microtarsus* and is one of the most well-characterized species in terms of mitochondrial bioenergetics.

Marsupials (*G. microtarsus*) were captured in the municipality of Américo Brasiliense,* ca* 300 km northwest of São Paulo in southeastern Brazil (collection permit number from the Brazilian Institute of Environment (IBAMA): SISBIO #36133). Vegetation at the location consists of forested remnants of Cerrado characterized by dense semideciduous forest with canopy cover varying from 50 to 90 percent, trees 8–15 m tall, and little herbaceous vegetation. The climate of the region has two well-defined seasons: a warm-wet season from October to March and a cool-dry season from April to September. Traps were set for four consecutive nights every month from February to November 2012. Animals were captured using a 11 × 8 trapping grid with 88 trapping stations located 10 m apart. A single Sherman live trap (7.5 × 9.0 × 23.5 cm) was set on trees at each trapping station* ca* 1.75 m aboveground and baited with banana, peanut butter, and cod-liver oil.

The marsupials (*G. microtarsus*) were returned to the Universidade Estadual de Campinas (UNICAMP) and housed in individual cages in an animal room maintained at approximately 23°C with a 12 h/12 h light/dark cycle. Marsupials were provided with* ad libitum* water and the appropriated amount of food (dry cat and dog food and mango) to maintain their weight gain similar to that expected under natural conditions. The individuals were kept in this animal room for approximately 3 months before the beginning of the experiments.

Experimental protocols used were approved by the local Committee for Ethics in Animal Research (CEUA-UNICAMP). Animal experiments followed the Guide for the care and use of laboratory animals published by the U.S. National Institutes of Health (NIH publication 85-23, revised 1996).

### 2.3. Isolation of Liver Mitochondria

Liver mitochondria were isolated concomitantly from mice and marsupials by differential centrifugation [25] and partially purified by a discontinuous Percoll gradient. Male animals were used for all experiments except that for quantification of mitochondrial NAD(P) contents. The animals were decapitated and the livers were rapidly removed, finely minced, and homogenized in an ice-cold isolation medium containing 250 mM sucrose, 1 mM EGTA, and 10 mM HEPES buffer (pH 7.2). The homogenates were centrifuged for 10 min at 800* g*. The supernatants were centrifuged at 7750* g* for 10 min. The mitochondrial pellet was purified using a discontinuous Percoll gradient according to Lopez-Mediavilla et al. [26]. After centrifugation for 10 min at 7750* g*, the mitochondrial fraction obtained from the interface between 19 and 52% Percoll layers was resuspended in buffer containing 250 mM sucrose, 0.3 mM EGTA, and 10 mM HEPES buffer (pH 7.2) and recentrifuged at 7750* g* for 10 min. The final pellet containing liver mitochondria was resuspended in an EGTA-free buffer at approximate protein concentrations of 50 mg/mL. The entire procedure was carried out at 4°C. The protein content of the mitochondrial suspensions was determined by Biuret assay in the presence of 0.2% deoxycholate [27] with bovine serum albumin as the standard.

### 2.4. Standard Incubation Procedure

Measurements of mitochondrial oxygen consumption, membrane potential, Ca^2+^ uptake, redox state of endogenous nicotinamide nucleotides, and activity of glutathione peroxidase/redutase system were carried out at 28°C with continuous magnetic stirring in a standard reaction medium containing 125 mM sucrose, 65 mM KCl, 2 mM KH_2_PO_4_, 1 mM MgCl_2_, 10 mM HEPES buffer (pH 7.2), and ~15 *μ*M contaminant Ca^2+^. Other additions are indicated in the figure legends. Except for the O_2_ consumption measurements, which were performed in a 1.4 mL chamber, a 2 mL final volume was used in the experiments that were performed in cuvettes.

### 2.5. Oxygen Consumption Measurements

Oxygen consumption by the mitochondria (0.5 mg/mL) was measured in a temperature controlled chamber equipped with a magnetic stirrer, using a Clark-type electrode (Yellow Spring Instruments Company, Yellow Spring, OH, USA) in standard reaction medium containing 0.3 mM EGTA and a NADH-linked substrate mixture (2 mM malate, 1 mM pyruvate, 1 mM *α*-ketoglutarate, and 1 mM glutamate).

### 2.6. Measurement of Transmembrane Electrical Potential

Mitochondrial membrane potential was monitored by following the changes in 5 *μ*M safranine fluorescence [28], which were recorded on a Hitachi F-4500 spectrofluorometer operating at excitation and emission wavelengths of 495 and 586 nm, respectively, with slit widths of 5 nm.

### 2.7. Measurements of Mitochondrial Ca^2+^ Retention Capacity

The Ca^2+^ retention capacity was determined in liver mitochondria (0.5 mg/mL) incubated in standard reaction medium containing 0.2 *μ*M Calcium Green-5N as a probe. Levels of external free Ca^2+^ were measured by recording the fluorescence of Calcium Green-5N on a spectrofluorometer (Hitachi F-4500) operating at excitation and emission wavelengths of 506 and 532 nm, respectively, with slit widths of 5 nm and continuous magnetic stirring. Five minutes after the addition of mitochondria (0.5 mg/mL) to the cuvette, boluses of 5 *μ*M (control conditions) or 30 *μ*M (when cyclosporin A, ADP, or ATP plus Mg^2+^ was present) of CaCl_2_ were sequentially added every 2.5 min until the mitochondria began to release Ca^2+^ into the medium. The amount of CaCl_2_ added prior to mitochondrial Ca^2+^ release was taken as the mitochondrial Ca^2+^ retention capacity, a quantitative approach to compare MPT between groups.

### 2.8. Determination of NAD(P) Redox State in Intact Mitochondria

Changes in the redox state of nicotinamide nucleotides (NAD(P)) in the mitochondrial suspensions (0.5 mg/mL) in standard reaction medium supplemented with 300 *μ*M EGTA, 1 *μ*M rotenone, and 5 mM succinate were monitored in a spectrofluorometer (Hitachi F-4500) using excitation and emission wavelengths of 366 and 450 nm, respectively, and slit widths of 5 nm [15]. Of note, only the reduced forms of NAD(P) exhibit a strong endogenous fluorescence signal. As a reference, known amounts of NADPH were added to the reaction medium in the absence of mitochondria. Succinate was chosen as an energizing substrate to allow the endogenous content of substrates, which was apparently different between species, to play a role in the metabolism of tert-butyl hydroperoxide (*t*-BOOH), an exogenous peroxide that was used to challenge the mitochondrial antioxidant system.

### 2.9. Mitochondrial Activity of Glutathione Peroxidase/Reductase System

Liver mitochondria (1 mg/mL) were lysed by the presence of 0.1% Triton X-100 in standard medium reaction containing 500 *μ*M GSH and 100 *μ*M NADPH. The activity of the mitochondrial glutathione peroxidase/reductase system was estimated by the rate of NADPH oxidation after the addition of 0.5 mM tert-butyl hydroperoxide (*t*-BOOH; an oxidant agent) [29]. NADPH oxidation was followed by monitoring the fluorescence at excitation and emission wavelengths of 366 and 450 nm, respectively, and slit widths of 5 nm. In this assay, added glutathione is recycled thought the action of both redox enzymes consuming the NADPH, thus revealing the maximal flux through this enzymatic system.

### 2.10. Nicotinamide Nucleotide Transhydrogenase (NNT) Assay

NNT was assayed as conducted before in our laboratory [15]. Briefly, the changes in differential absorbance (375–425 nm) due to the reduction of APAD, which is a NAD^+^ analogue, were monitored for 5 min at 37°C (Shimadzu UV-1800 Spectrophotometer, Kyoto, Japan). The assay medium contained 100 mM sodium phosphate (pH 6.5), 1 mg/mL lysolecithin, 0.5% Brij-35, 1 *μ*M rotenone, 300 *μ*M APAD, and 400 *μ*g/mL liver mitochondrial protein; the reaction was initiated with 300 *μ*M NADPH after 5 min preincubation. The slopes of absorbance over time were converted to nmol APAD reduced/min using the molar extinction coefficient of 5.1 mM^−1^ × cm^−1^ for reduced APAD.

### 2.11. Quantification of Mitochondrial NAD(P) Contents

Oxidized and reduced forms of NAD and NADP were determined by fluorometric detection using high-performance liquid chromatography (HPLC) as described by Klaidman et al. [30] with minor modifications [15]. Calibration curves were built with known amounts of standards. All samples concomitantly isolated from marsupials and mice were immediately frozen and maintained at −80°C until analysis a week later.

### 2.12. Statistics

Results are presented as representatives or averages ± standard errors (SEM) of at least three experiments with different preparations. Mann-Whitney (nonparametric) test or Student's *t*-test was used for statistical analyses. A *P* value less than 0.05 was considered significant.

## 3. Results 

### 3.1. Respiratory Coupling

**Figure 1:**
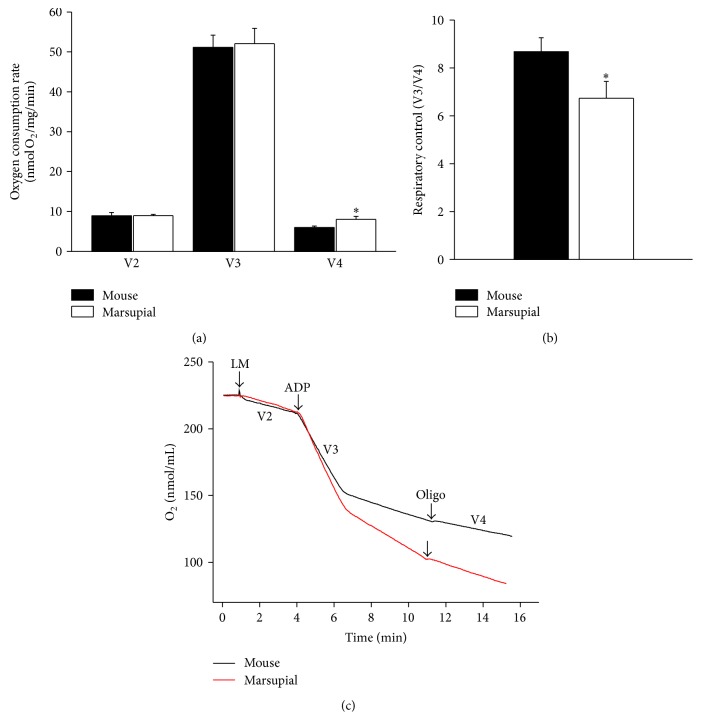
Oxidative phosphorylation parameters in mouse and marsupial liver mitochondria. (a)* Mus musculus* (mouse) and* Gracilinanus microtarsus* (marsupial) isolated liver mitochondria (0.5 mg/mL) were incubated in standard reaction medium containing NADH-linked respiratory substrates (2 mM malate, 1 mM pyruvate, 1 mM *α*-ketoglutarate, and 1 mM glutamate) and 200 *μ*M EGTA. Respiratory states were determined under basal conditions (V2) and after sequential additions of ADP (300 *μ*M) and 1 *μ*g/mL oligomycin (Oligo) to achieve respiratory states 3 (V3) and 4 (V4), respectively. The results are expressed as means ± SEM (*n* = 8). ^*∗*^ *P* < 0.05 versus mouse mitochondria. (b) Respiratory control ratios (V3/V4). (c) Representative traces of oxygen consumption by mouse and marsupial liver mitochondria (LM).

In order to assess the functional integrity of isolated mitochondrial preparations, respiration experiments were performed (Figures 1(a)–1(c)). Both marsupial and mouse liver mitochondria demonstrated well-coupled respiration although the respiration in the presence of oligomycin (state 4 respiration; V4) was significantly higher in the marsupial liver mitochondria. The mean respiratory control ratio (RCR) was slightly higher in mice than in marsupial.

### 3.2. Electrical Membrane Potential (ΔΨ): Effect of Ca^2+^

**Figure 2:**
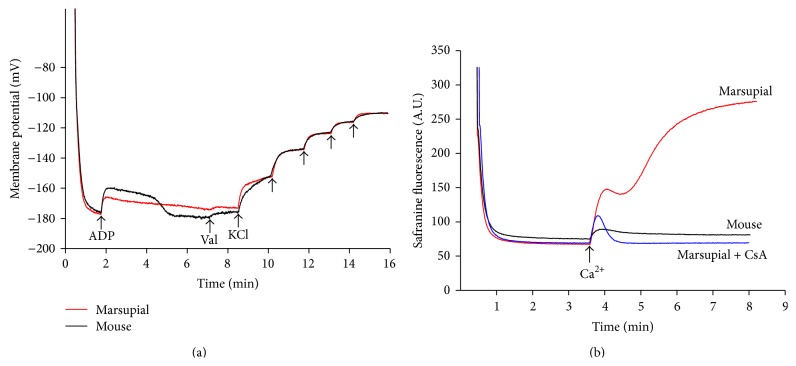
Effect of micromolar Ca^2+^ concentration on membrane potential of mouse and marsupial liver mitochondria. (a) Determination of membrane potential in isolated mouse and marsupial liver mitochondria. Liver mitochondria (0.5 mg/mL) were added to the incubation medium containing 200 *μ*M EGTA, NADH-linked respiratory substrates (2 mM malate, 1 mM pyruvate, 1 mM *α*-ketoglutarate and 1 mM glutamate), and 5 *μ*M safranine. The arrows indicate where 150 *μ*M ADP, 40 ng/mL valinomycin (Val), and KCl (each addition: 350 *μ*M) were added to the experiments. (b) Effect of Ca^2+^ on mitochondrial membrane potential. Ca^2+^ (40 *μ*M) was added to the experiments which was indicated by the arrow. Cyclosporin A (CsA; 1 *μ*M), a mitochondrial permeability transition inhibitor, was present in the incubation medium where indicated. Traces are representative of 3 independent experiments.

The experiment depicted in Figure 2 demonstrated that energization of both types of mitochondria was followed by safranine uptake and adsorption to the polarized inner membrane, processes associated with safranine fluorescence decrease [28]. It can be observed that the initial decreases in fluorescence were quantitatively similar in both mitochondria and stabilized at membrane potentials close to −180 mV (Figure 2(a)). ADP addition to mouse liver mitochondria induced the expected transient decrease in ΔΨ, returning to the previous value after a short period of ADP phosphorylation. ADP addition to the marsupial mitochondria also caused the expected ΔΨ decrease with a slow return to initial values. ΔΨ was estimated by calibration through potassium titration after the ionophore valinomycin was included in the medium [28].

Interestingly, Figure 2(b) shows that the marsupial mitochondria quickly released the ΔΨ after the addition of a small pulse of Ca^2+^ (30 *μ*M) via a mechanism sensitive to cyclosporin A. In contrast, mouse liver mitochondria sustained a very stable membrane potential after the transient decrease in ΔΨ induced by the same pulse of Ca^2+^.

### 3.3. Ca^2+^ Retention Capacity

**Figure 3:**
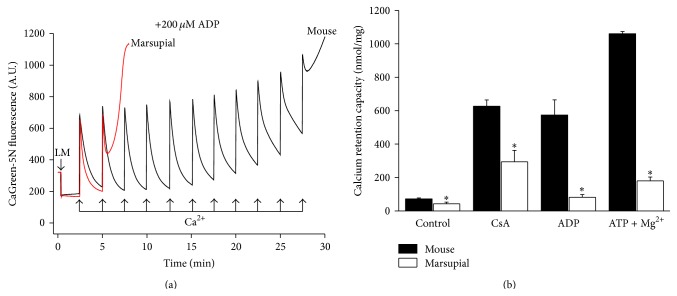
Effect of mitochondrial permeability transition (MPT) inhibitors on the Ca^2+^ retention capacity of mouse and marsupial liver mitochondria. Isolated mouse and marsupial liver mitochondria (LM; 0.5 mg/mL) were incubated in standard reaction medium supplemented with NADH-linked (2 mM malate, 1 mM pyruvate, 1 mM *α*-ketoglutarate, and 1 mM glutamate) respiratory substrates and 0.2 *μ*M Calcium Green-5N. Some experiments were conducted in the presence of the MPT inhibitors: 1 *μ*M cyclosporin A (CsA), 200 *μ*M ADP, or 200 *μ*M ATP plus 3 mM MgCl_2_, as indicated in the figure. (a) Representative experiments for estimation of calcium retention capacity of mouse and marsupial liver mitochondria in the presence of ADP. (b) To assess the mitochondrial Ca^2+^ retention capacity, pulses of Ca^2+^ (5 *μ*M for control conditions or 30 *μ*M for the conditions in the presence of MPT inhibitors CsA, ADP, or ATP plus Mg^2+^) were added until mitochondrial Ca^2+^ release occurred. The sum of Ca^2+^ pulses prior to MPT pore opening was taken as the mitochondrial Ca^2+^ retention capacity (*n* = 7 for all conditions except for “ATP+Mg^2+^,” where *n* = 3). ^*∗*^ *P* < 0.05 versus respective condition in mouse mitochondria.

Considering that Ca^2+^-induced MPT is an event that is redox sensitive and may promote cell death [4, 13], we determined the mitochondrial Ca^2+^ retention capacity of both types of mitochondria as an assessment of their susceptibility to MPT.  Figure 3(a) depicts representative experiments of mitochondria oxidizing NAD-linked substrates, in the presence of ADP, and subjected to successive additions of Ca^2+^ pulses, to the point of MPT-mediated Ca^2+^ release. It can be seen that the marsupial liver mitochondria exhibited a significantly lower Ca^2+^ retention capacity than mouse mitochondria. Although the known MPT inhibitors cyclosporin A, ADP, and ATP plus Mg^2+^ [12, 13, 31, 32] significantly increased the capacity of the marsupial mitochondria to retain the cation, their effects were much larger in mice mitochondria (Figure 3(b)). For example, in the presence of ADP the capacity of Ca^2+^ retention by the marsupial liver mitochondria was almost ten times lower than that of the mice liver mitochondria.

### 3.4. Mitochondrial Nicotinamide Nucleotide Content and Redox State

**Figure 4:**
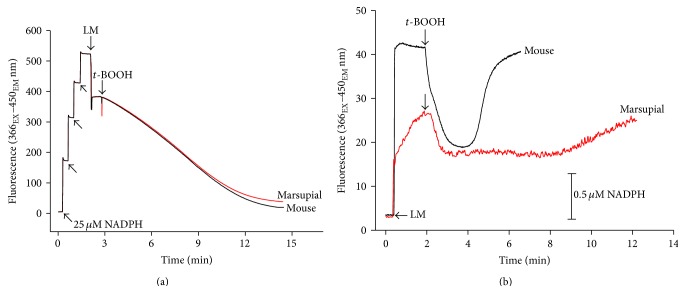
Determination of the mitochondrial activity of glutathione peroxidase/reductase system and ability to metabolize organic peroxide. (a) Isolated mouse and marsupial liver mitochondria (LM; 1 mg/mL) were added to standard reaction medium containing 500 *μ*M GSH and 100 *μ*M NADPH. Triton X-100 (0.1%) was also presented to lyse the mitochondria. The reaction started after the addition of 0.5 mM tert-butyl hydroperoxide (*t*-BOOH). Lines are representative of three independent experiments. (b) LM (0.5 mg/mL) were incubated in standard medium containing 5 mM succinate, 1 *μ*M rotenone, and 300 *μ*M EGTA. Where indicated by the arrow, 15 *μ*M* t*-BOOH was added. Traces are representative of 4 independent experiments.

It has long been known [33] that the reduced state of mitochondrial nicotinamide nucleotides, mainly NADPH [14], favors Ca^2+^ retention by mitochondria. To assess the participation of these nucleotides in these mechanisms, we monitored fluorimetrically the changes in redox state of mitochondrial NAD(P)H during the detoxification of exogenously added tert-butyl hydroperoxide (*t*-BOOH) in both types of mitochondria. Firstly we analyzed the activity of the glutathione peroxidase/reductase system that catalyzes this reaction using reducing equivalents from NADPH [29].  Figure 4(a) provided evidence that the activities of these enzymes are quite similar in both marsupial and mice mitochondria but the results presented in Figure 4(b) indicated that (i) upon the addition of mitochondria to the reaction medium, the mice nicotinamide nucleotides fluorescence was at the maximum value and maintained a plateau while the marsupial nicotinamide nucleotides were not at the maximal value but steadily increased their fluorescence toward a lower plateau value than that of the mice nicotinamide nucleotides fluorescence, (ii) the extent of the redox changes induced by* t*-BOOH was much smaller in marsupial mitochondria, and (iii) the time to restore the* t*-BOOH induced NAD(P)H oxidation was much longer in marsupial mitochondria. Taken together these results indicate that both content and state of reduction were lower in the marsupial mitochondria than in mice mitochondria. Under the conditions with succinate as an energy substrate that was used to obtain data shown in Figure 4(b), the function of NNT is required (as demonstrated in [15]) to support NADP^+^ reduction. For this reason and because the rereduction of NAD(P) was much slower in marsupial than in mouse mitochondria following* t*-BOOH addition, we assayed NNT activity in isolated liver mitochondria from both species. The measured activity of NNT was not different between marsupial (28.0 ± 1.12 mU/mg; *n* = 3) and mouse (30.9 ± 5.02 mU/mg; *n* = 3), thus ruling out the involvement of NNT activity in the slower peroxide metabolism by marsupial mitochondria compared to that of mouse.

**Figure 5:**
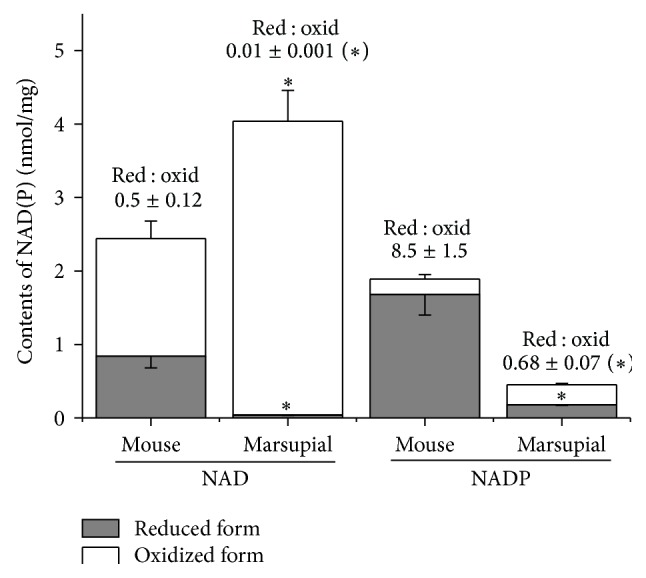
Determination of nicotinamide nucleotide content in isolated mitochondria. HPLC analysis of NAD and NADP contents in mouse and marsupial mitochondria (*n* = 3 for mouse and 5 for marsupial). The ratio of reduced to oxidized NAD(P) (Red : Oxid) is shown above the bars. ^*∗*^ *P* < 0.05 versus respective parameter in mouse mitochondria.

In order to further investigate the redox state and the content of nicotinamide nucleotides in mitochondria, we performed HPLC analysis of these nucleotides. The bars presented in Figure 5 show that the content of total NAD is higher in the marsupial than in mice mitochondria (4.02 ± 0.42 versus 2.44 ± 0.62 nmol/mg) and that, in contrast, the content of total NADP is much lower in the marsupial mitochondria (0.44 ± 0.03 versus 1.89 ± 0.32 nmol/mg). Most interesting, and in agreement with the data presented in Figure 4(b), both nicotinamide nucleotides were much more oxidized in the marsupial mitochondria. Overall, data in Figures 4(b) and 5 seem to indicate that marsupial liver mitochondria possess a lower content of endogenous substrates linked to NAD(P)^+^ reduction than that of mice.

## 4. Discussion 

Mitochondrial dysfunction and opening of the PTP are thoroughly implicated in the development of several diseases and aging, in various animal models [4, 11–13]. In this regard, the present work demonstrates that isolated liver mitochondria from the short life span marsupial* G. microtarsus* presented three main functional differences when compared to mice mitochondria, which were used here as an established mammalian model, for comparative purposes. First, the marsupial mitochondria showed a significant higher rate of resting (state-4) respiration; second, they are much more susceptible to PTP opening; and third, they have a much lower constitutive antioxidant capacity represented by the NADPH/NADP^+^ content and redox potential.

The higher state-4 respiration was present in all marsupial liver mitochondrial preparations as compared to mouse. From the stand point of redox regulation of MPT that will be approached below, it might be worth mentioning that higher mitochondrial respiration rates are associated with lower rates of superoxide radical production by mitochondria [4]. Since the most frequently used technique to assess ROS production from mitochondria (Amplex Red/horseradish peroxidase assay) may not be suitable to compare isolated liver mitochondria from different species [34], we performed analyses of the mitochondrial antioxidant system. These evaluations indeed revealed main mitochondrial redox differences between these two species with regard to the regulation of MPT. It might be speculated that the higher state-4 respiration in marsupial could be linked to an impaired clearance process of old or damaged mitochondria that may contribute to a fast process of senescence related to the short life span of this marsupial [35, 36]. Except for this presumed subpopulation of uncoupled mitochondria and the higher susceptibility to Ca^2+^-induced MPT the marsupial and mice liver mitochondria exhibited similar bioenergetics properties when evaluated under the same experimental conditions. As shown in the results section, liver mitochondria from both species were well coupled and presented comparable values of electrical membrane potential and maximal rates of ADP-stimulated respiration. Therefore, the difference in susceptibilities to MPT cannot be attributed to differences in quality between the two mitochondrial preparations. Indeed, research in progress in this laboratory provides evidence that fish liver mitochondria present lower respiratory control ratio and higher state-4 respiration than rat liver mitochondria; but in contrast to these marsupial mitochondria, fish liver mitochondria have a much higher capacity to retain Ca^2+^ than rat (G. A. Dal'Bó, F. G. Sampaio, A. E. Vercesi, unpublished results). In fact, the present experiments demonstrate that marsupial and mice mitochondria share some MPT properties and differ in some other properties. The results depicted in Figure 3 indicate that the marsupial mitochondria present a lower threshold for Ca^2+^ induced PTP opening. However, it should be emphasized that even when PTP is inhibited by CsA the ability of the marsupial mitochondria to accumulate and retain Ca^2+^ is significantly lower than that of the mice mitochondria. In addition, MPT in the marsupial mitochondria is less sensitive to the inhibition by adenine nucleotides ATP or ADP, especially to the latter. For example, while the mice mitochondria accumulated and retained ten pulses of 60 nmol Ca^2+^/mg before opening the PTP in the presence of ADP, marsupial mitochondria were able to accumulate and retain only one pulse (Figure 3(a)).

Despite the large number of studies approaching the PTP structure, its composition remains unresolved and controversial. Several studies suggest that it is minimally composed of or modulated by matrix, inner and outer membrane proteins such as the CsA-binding protein cyclophilin D (CypD), the adenine nucleotide transporter (ANT), the ATP synthase, hexokinase, phosphate carrier, and the voltage dependent anion channel (VDAC) (for recent reviewers see [37, 38]). Other studies using submitochondrial particles, mitoplasts, or mitochondria naturally or genetically modified provided evidence that PTP opening may take place although with different characteristics even in the absence of some of these proteins [39–43]. Therefore, the present results showing different properties between marsupial and mice PTP can be interpreted taking into consideration the different plasticity and protein composition of the putative PTP pore. In addition, oxidative stress may also contribute to the lower inhibitory effect of adenine nucleotides on Ca^2+^ induced MPT [31] in marsupial mitochondria.

Perhaps the most intriguing characteristic of the marsupial liver mitochondria is their low capacity for Ca^2+^ retention. This recall pioneering data from Lehninger laboratory demonstrating that Ca^2+^ release from liver mitochondria was favored by the oxidized state of endogenous nicotinamide nucleotides [33]. The progress in the understanding of these data provided evidence that PTP opening is associated with membrane protein thiol crosslinking via thiol oxidation linked to the redox state of mitochondrial NADP [39]. In fact, MPT can be stimulated in Ca^2+^ loaded mitochondria by prooxidants such as *t-*BOOH, diamide, suramin, and/or by various experimental conditions that lead to oxidative stress either in isolated mitochondria, intact cells, or isolated organs [13, 14, 44, 45].

In the present work, a more oxidized state of the mitochondrial NADP was demonstrated by HPLC analysis which strongly supports the idea that the mitochondrial NADPH-dependent antioxidant systems glutathione and thioredoxin peroxidases/reductases are less effective in the marsupial due to a lower reducing power provided by NADPH (Figure 4(a)). This hypothesis was further corroborated by the experiment depicted in Figure 4(b) showing a much slower rate of *t*-BOOH metabolization by the marsupial than by the mouse liver mitochondria. This is also in agreement with the recent studies from this laboratory showing that liver mitochondria isolated from the expontaneously mutated C57BL/6J mice lacking functional mitochondrial nicotinamide nucleotide transhydrogenase (NNT), an enzyme that reduces NADP^+^ using reducing equivalents from NADH, are more susceptible to MPT [15]. Although the marsupial and the NNT-mutated mice exhibit a compromised NADPH reducing power via different mechanisms, they share common mitochondrial characteristics namely low antioxidant mitochondrial capacity and high susceptibility to MPT.

A complex phenotype, as the short life span of this marsupial, may be determined by the interaction of many variables, among which the observed mitochondrial characteristics may comprise an intrinsic biochemical factor reducing survival upon environmental challenges.

## Abbreviations

ADP: Adenosine diphosphate
ATP: Adenosine triphosphate
CsA: Cyclosporin A
KCl: Potassium chloride
LM: Liver mitochondria
MPT: Mitochondrial permeability transition
NAD: *β*-Nicotinamide adenine dinucleotide
NADH: Reduced form of NAD
NAD^+^: Oxidized form of NAD
NADP: *β*-Nicotinamide adenine dinucleotide phosphate
NADPH: Reduced form of NADP
NADP^+^: Oxidized form of NADP
NNT: Nicotinamide nucleotide transhydrogenase
Oligo: Oligomycin
PTP: Permeability transition pore
ROS: Reactive oxygen species
*t*-BOOH: tert-Butyl hydroperoxide
Val: Valinomycin.
